# A model for the easy assessment of pressure-dependent damage to retinal ganglion cells using cyan fluorescent protein–expressing transgenic mice

**Published:** 2012-10-05

**Authors:** Hidekazu Tsuruga, Hiroshi Murata, Makoto Araie, Makoto Aihara

**Affiliations:** Department of Ophthalmology, University of Tokyo School of Medicine, Tokyo, Japan

## Abstract

**Purpose:**

To establish an animal model for the easy assessment of pressure-dependent damage to retinal ganglion cells (RGCs) using the B6.Cg-TgN(Thy1-CFP)23Jrs/J transgenic mouse strain (CFP mouse), which expresses cyan fluorescent protein (CFP) in RGCs, and to evaluate pressure-dependent RGC death.

**Methods:**

In 20 CFP mice, right eyes were selected to receive laser-induced ocular hypertension eye and the contralateral eyes remained untouched to serve as controls. Intraocular pressure (IOP) was measured each week in both eyes using the microneedle method up to 8 weeks. Based on the line plot of time (*x*) and IOP (*y*) in laser-treated and control eyes, the area surrounded by both lines (∫*Δ*IOP(*y*) d*x*) was calculated as a surrogate value of the pressure insult. At 9 weeks, eyes were enucleated and RGCs expressing CFP were evaluated histologically in retinal whole mounts. The correlation between pressure insult and RGC density was evaluated in the whole eye, three concentric regions, and four quadrants.

**Results:**

Laser-treated eyes showed a significantly higher IOP than control eyes from 1 to 7 weeks (p<0.01). The pressure insult and the RGC density showed a significant negative correlation (y=-0.070x+97.2, r=0.75, p=0.0008). Moreover, the central, middle, and peripheral areas measured from the optic disc and each of four retinal quadrant areas also showed significant negative correlations. Our data demonstrate that each retinal area was almost evenly damaged by IOP elevation.

**Conclusions:**

Laser photocoagulation causes a chronic elevation of IOP in CFP mice. The use of CFP mice enabled us to easily evaluate pressure-dependent RGC damage. This glaucomatous CFP mouse model may contribute to the molecular analysis of neurodegeneration and the development of neuroprotective drugs for glaucoma with a great increase in experimental efficiency.

## Introduction

Glaucoma elicits a progressive visual field defect with loss of retinal ganglion cell (RGC) axons, which is known as glaucomatous optic neuropathy (GON) [1]. Elevated intraocular pressure (IOP) is considered to be a major risk factor for the development and progression of glaucoma [2]. Additionally, it has been hypothesized that various risk factors of GON, such as hypoxia, glutamate stress, oxidative stress, or genetic factors, are related to the pathogenesis of GON. Moreover, molecular mechanisms of apoptotic RGC death have also been investigated in relation to preventing stress-induced RGC death [3-6]. New therapeutic modalities targeting RGC survival against various stresses, including elevated IOP, have been assessed. To date, various animal models mimicking GON by increasing IOP or inducing various stresses have been developed to screen neuroprotective drugs [7,8]. Obviously, the most effective and quantitative screening system will be able to determine the results of a drug effect in a short time period with a small number of animals.

Previous investigations of the mechanism of GON have used animals with experimentally induced ocular hypertension. In particular, mice have great advantages as they are relatively cheap and easily handled; moreover, there are various knockout or transgenic strains available to clarify the molecular mechanisms of GON [9]. However, the small size of the eye and the difficulty in obtaining IOP data has been a great challenge in using mouse eyes in glaucoma studies.

RGCs were labeled in a retrograde fashion with dyes injected from the superior colliculus, and counted [10]. However, RGCs may behave abnormally due to unexpected insult with injected dye or cranial surgery. Recently, B6.Cg-TgN(Thy1-CFP)23Jrs/J transgenic mice, which express cyan fluorescent protein (CFP) in RGCs in the eye [11,12], have been used to visualize GON. The use of glaucomatous CFP mice enables the easier evaluation of glaucomatous neuropathy without the retrograde labeling of RGCs.

Our group and Leung et al. reported that a prospective and noninvasive evaluation procedure that involves taking fundus photographs of the CFP mouse retina is useful to evaluate RGC death more effectively and quantitatively [13,14]. Following these reports, RGC death by optic nerve crush and ischemia reperfusion has been evaluated using the CFP mouse [15-18]. However, IOP-dependent RGC death has not been clarified using the CFP mouse. Because increasing and monitoring IOP is especially difficult in small animals, the establishment of an ocular hypertension model mouse is still a great challenge. Although Tosi et al. also reported RGC damage in DBA2J ocular hypertension mice by mating this strain with the CFP mouse, the correlation between IOP insult and RGC damage was not clarified [16].

Our group previously reported a laser-induced ocular hypertension mouse model using the conventional C57BL6 mouse strain [19], and subsequently reported regional optic nerve damage, indicating that the superior quadrant was particularly susceptible to damage in the mouse [20]. In the latter report, optic nerve axons were histologically analyzed and counted in electron microscopic images. In spite of a great deal of effort, only 5% of axons were counted and the correlation between IOP elevation and axon damage was not assessed.

In this study, we have established a laser-induced ocular hypertensive CFP mouse model with careful monitoring of IOP. Moreover, we quantitated pressure-dependent RGC damage to use our mouse model for the evaluation of neuroprotective drugs.

## Methods

### Animals

The animals used in this study were treated in accordance with the ARVO Statement for the Use of Animals in Ophthalmic and Vision Research. Male C57BL/6J mice were purchased from Japan SLC (Hamamatsu, Japan). Male and female B6.Cg-TgN(Thy1-CFP)23Jrs/J mice, in which the Thy1 promoter is linked to a CFP reporter, were obtained from the breeding colony of the Jackson Laboratory (Bar Harbor, ME). Mice were bred and housed in clear cages covered loosely with air filters. The cages contained white chip bedding. The environment was kept at 21 °C with a 12 h:12 h light-dark cycle. All mice were fed ad libitum. In all experiments, we used mice aged 16 to 24 weeks old.

### Induction of experimental ocular hypertension

**Figure 1:**
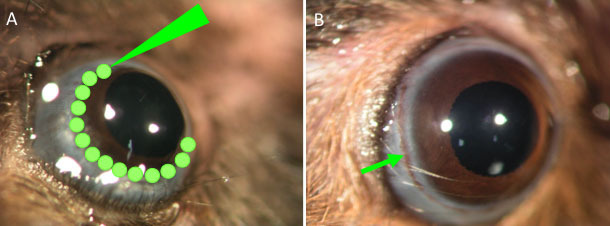
Laser procedure to increase intraocular pressure. **A**: Green spots indicate the scheme of laser photocoagulation performed to increase intraocular pressure (IOP) around the whole circumference of the limbus after flattening the anterior chamber by aspiration of aqueous humor. **B**: The laser-treated eye of one animal at 8 weeks after laser photocoagulation indicated high IOP. Arrow indicates laser spots.

Aqueous outflow in the right eye was obstructed by laser photocoagulation at the corneal limbus, as described previously [19]. Briefly, the right eyes received laser-induced ocular hypertension and the contralateral eyes remained untouched to serve as controls. After anesthesia was administered with an intraperitoneal injection of a solution containing ketamine (100 mg/kg) and xylazine (9 mg/kg) and pupils were dilated by instillation of 5 μl of 0.5% tropicamide and 0.5% phenylephrine hydrochloride (Mydrin-P; Santen Pharmaceutical, Osaka, Japan), laser photocoagulation (532 nm wavelength, 200 mW power, 0.05 s duration, 200 μm spot size) was performed. After flattening the anterior chamber by aspiration of aqueous humor, 50–63 laser shots over a 270° circumference of the limbus were applied (Figure 1). To prevent potential infection, an antibiotic ointment was applied topically after the procedure. Additionally, a drop of 0.01% betamethasone was applied topically for 7 days to prevent inflammation.

### Measurement of intraocular pressure

The IOPs of CFP mice were measured every week for 8 weeks using the microneedle method, as previously described [19]. Briefly, a microneedle made of borosilicate glass (75–100 μm tip diameter and 1.0 mm outer diameter; World Precision Instruments, Sarasota, FL) was connected to a pressure transducer (model BLPR; World Precision Instruments). The system pressure detected by the transducer was recorded by a data acquisition and analysis system (PowerLab; AD Instruments, Colorado Springs, CO). The microneedle was placed in the anterior chamber and the conducted pressure was recorded in both eyes after anesthesia.

### Calculation of pressure insult

**Figure 2:**
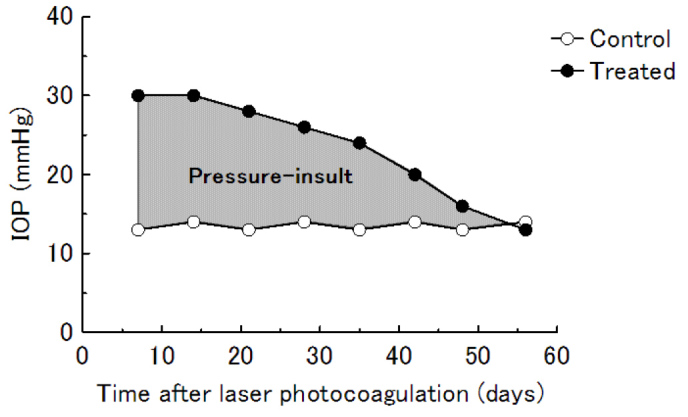
Definition of pressure-insult for a surrogate value. Based on the line plot of time (x) and IOP (y) in laser-treated and control eyes, the area surrounded by both lines (∫ΔIOP(y) dx) was calculated as a surrogate value of the pressure-insult.

The IOPs in the treated and the contralateral control eyes and the duration of IOP elevation in the treated eye were calculated. For each animal, a graph of IOP over time was constructed for the treated and control eyes. The area under the curve of the treated and control eyes was calculated in units of mmHg×day. The area under the control eye curve was subtracted from the area under the treated eye curve to assess the magnitude of IOP elevation. Total area for the period during which the IOP in the treated eye was higher than in the control eye defined a surrogate value of the pressure insult in the treated eye for elevated IOP (Figure 2).

### Retinal ganglion cell count

**Figure 3:**
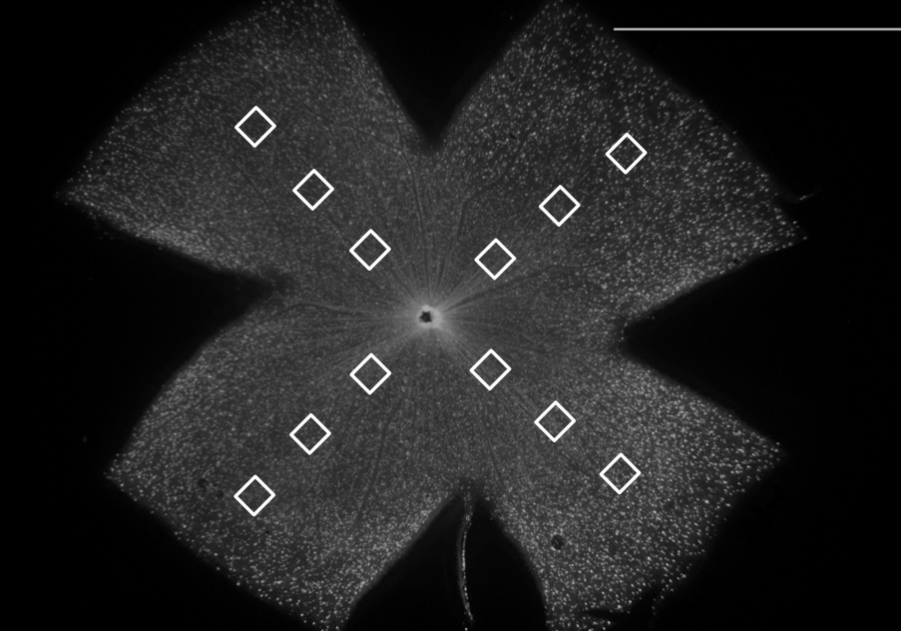
Counted areas in the whole mount retina. Squares of 200×200 µm were counted at 600 μm (central), 1200 μm (middle), and 1800 μm (peripheral) from the optic disc in each of four retinal quadrants. Scale bar indicates 2 mm.

Nine weeks after laser photocoagulation, the mice were sacrificed. The eyes were enucleated and immediately fixed in 4% paraformaldehyde in 0.1M phosphate buffered saline (PBS, 0.1M, pH 7.4, Muto Pure Chemicals Co. Ltd. Tokyo, Japan) for 1 h at 4 °C. Four radial relaxing incisions were made and the retina was prepared as a flattened whole mount on a glass slide with a coverslip. Images were obtained using a fluorescence microscope (BX50: Olympus, Tokyo, Japan) with a CFP filter set. The number of RGCs expressing CFP was manually counted using image-processing software (ImageJ) in 12 separate areas of 40,000 μm^2^. Areas that were 600 μm (central), 1200 μm (middle), and 1800 μm (peripheral) away from the optic disc in each of four retinal quadrants were sampled (Figure 3). The average density of RGCs/mm^2^ for each retinal area was obtained. The identity of the digitized images was masked before analysis. The RGC survival rate in the laser-treated eye was calculated as a percentage of RGC density in the contralateral control eye. The correlation between pressure insult and RGC survival rate was assessed by regression analysis.

### Histological evaluation

After the last IOP measurement, four mice were chosen for histological evaluation. Nine weeks after laser photocoagulation, the mice were sacrificed; the eyes were enucleated and immediately fixed in 4% paraformaldehyde and 2.5% glutaraldehyde in 0.1M PBS. The globes were embedded in paraffin and sequential meridian sections (10 μm thickness) were made through the optic disc. Sections were stained with hematoxylin and eosin, and examined with a light microscope (BX50; Olympus).

### Investigation of acute intraocular pressure elevation after laser photocoagulation

We used two methods to measure the IOP of C57BL/6J mice, specifically the microneedle method and TonoLab. Laser-induced ocular hypertension was elicited in the right eyes, as described above. The contralateral eyes received the same number of laser spots exteriorly over the 270° range of the limbal circumference as a sham operation without flattening of the anterior chamber. There was no induction of ocular hypertension in our preliminary studies (data not shown). The IOPs at 1, 2, 3, and 4 h following laser photocoagulation were assessed using a TonoLab calibrated for mice and validated in previous studies [21]. TonoLab provides similar readings to the microneedle method [22,23]. Three consecutive IOP readings in TonoLab were averaged per eye. The IOPs at 12, 24, 48, and 72 h following laser photocoagulation were measured by the microneedle method. All IOP data were analyzed by averaging IOP measurements at each time point.

### Statistical analysis

The paired *t* test and linear regression analysis were used for evaluation of the study results. A linear mixed model was used to compare the correlation coefficients between pressure insult and RGC survival rate in each retinal area, and p<0.05 was considered statistically significant. All data are presented as mean±standard deviation (SD).

## Results

### Induction of experimental ocular hypertension

**Figure 4:**
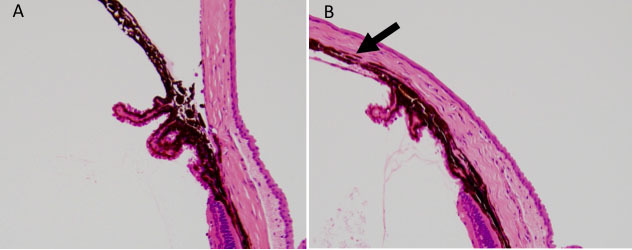
Histological assessment of the eye with ocular hypertension. Anterior segment of laser-treated **B**: and control **A**: eye was histologically assessed. The arrow indicates the closed angle induced by laser photocoagulation.

Twenty mice among those treated by laser photocoagulation survived over 9 weeks. Laser spots were observed over the episcleral vein regions in the laser-treated eyes (Figure 1). Histological analysis of paraffin-embedded eye sections showed that the angle of the laser-treated eye was completely closed (Figure 4). Neither the outer segments nor the retinal pigment epithelium of the peripheral retina exhibited signs of damage induced by direct application of the laser (data not shown).

**Figure 5:**
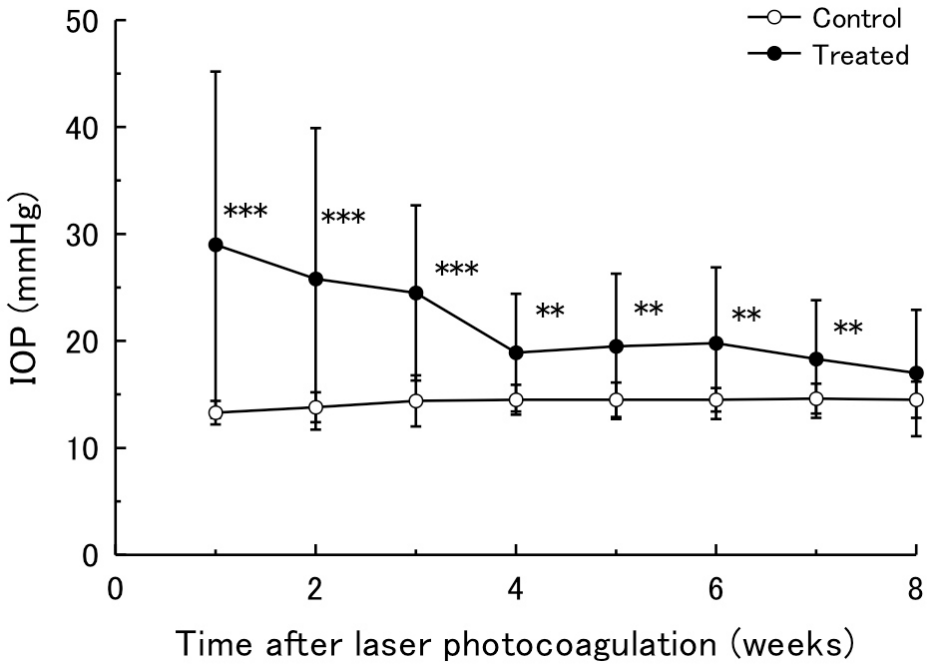
Intraocular pressure of ocular hypertension model eyes. Time courses of IOP in the laser-treated eyes and the contralateral control eyes for 8 weeks are indicated. n=20. Data are mean±standard deviation (SD). The IOP of the treated eyes was significantly higher in each time point compared with the IOP of the sham-operated contralateral eyes by a paired *t* test. (**p<0.01, *** p<0.001).

IOP elevation and duration were different in each eye. Sixteen of 20 treated eyes indicated over 30% IOP elevation for at least one measurement time point. However, 4 of 20 treated eyes indicated less than 30% elevation of IOP. The average IOP of the laser-treated eyes was 29.0±16.2 mmHg (mean±SD) at 1 week, which was markedly elevated compared with that of the nontreated control eyes (control, 13.3±1.1 mmHg). IOP remained significantly higher than that of the control eyes until 7 weeks after laser treatment (p<0.01). The average IOP of control eyes was stable and ranged from 13.3±1.1 to 14.6±1.4 mmHg during the entire 8 week study period. Sixteen (80%) of the 20 treated eyes had more than a 30% IOP increase at 1 week after treatment. At 4 and 8 weeks, 11 (55%) and 7 (35%) of 20 eyes maintained more than a 30% IOP increase and the mean IOPs were 18.9±5.5 mmHg and 17.0±5.9 mmHg, respectively (Figure 5).

### Investigation of acute intraocular pressure elevation after laser photocoagulation

**Figure 6:**
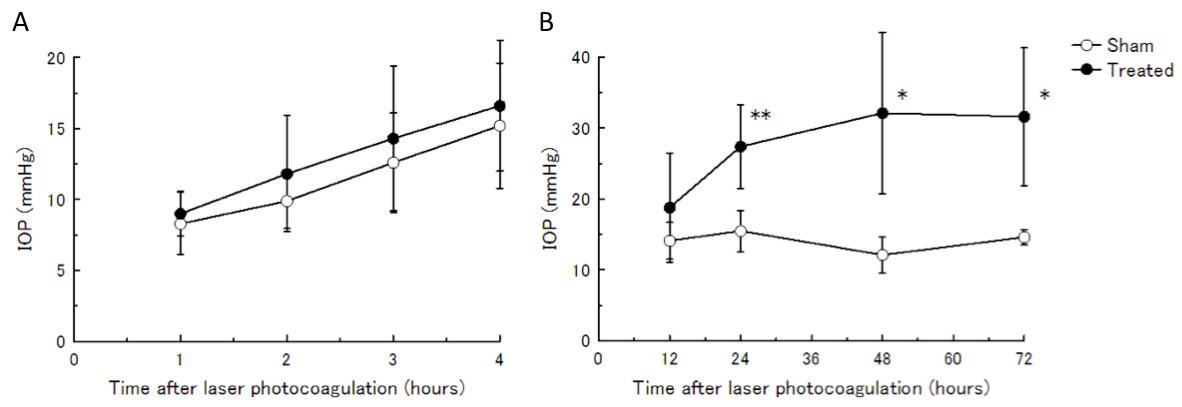
IOP elevation in the acute stage after laser photocoagulation. **A**: IOPs at 1, 2, 3, and 4 h after laser photocoagulation were measured using a TonoLab. **B**: IOPs at 12, 24, 48, and 72 h after laser photocoagulation were measured by the microneedle method. Data are mean±standard deviation (SD). n=5–6. The IOP of the treated eyes was significantly higher in each time point compared with the IOP of the sham-operated contralateral eyes by a paired *t* test. (*p<0.05, ** p<0.01).

To evaluate whether acute IOP elevation occurred after laser photocoagulation, we measured early IOP changes during the first 1–72 h. For the first 4 h, both the laser-induced ocular hypertension eyes and the sham-operated contralateral eyes gradually increased in IOP. However, the laser-induced ocular hypertension eyes showed a significant IOP elevation from 24 h to 72 h after laser photocoagulation compared with the IOP of sham-operated eyes without flattening of the anterior chamber (p<0.05; Figure 6). There was no significant difference from 1 h to 12 h. The IOP of sham-operated C57BL6/J eyes was 15.2 mmHg after 4 h, which is similar to that of nontreated eyes in a previous report [24,25]; this IOP was stable for 72 h. Consequently, the investigation revealed that the IOP of laser-treated eyes gradually increased until 24 h and sustained ocular hypertension.

### Histological analysis of retinal ganglion cells and the optic disc

**Figure 7:**
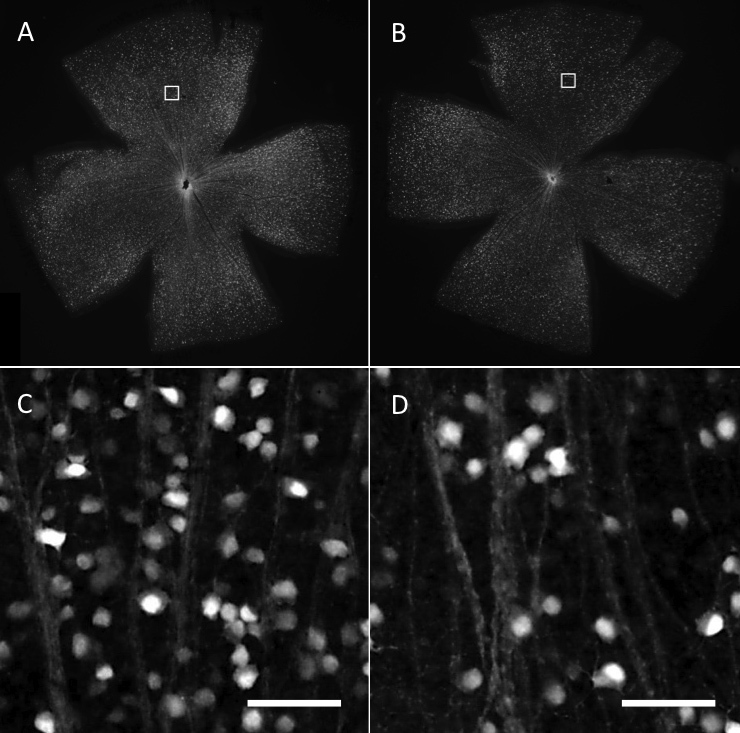
RGC images expressing cyan fluoresceint protein (CFP) in the retina. Whole mount retinal images of control eye **A**: and laser-treated eye **B**: were taken under the fluorescein microscope. Magnified images corresponding to the white box in **A** and **B**: at 1200 μm from the optic disc in the superior area are **C**: and **D**: The numbers of RGC expressing CFP decreased in the laser-treated eye. Scale bar is 50 μm.

**Figure 8:**
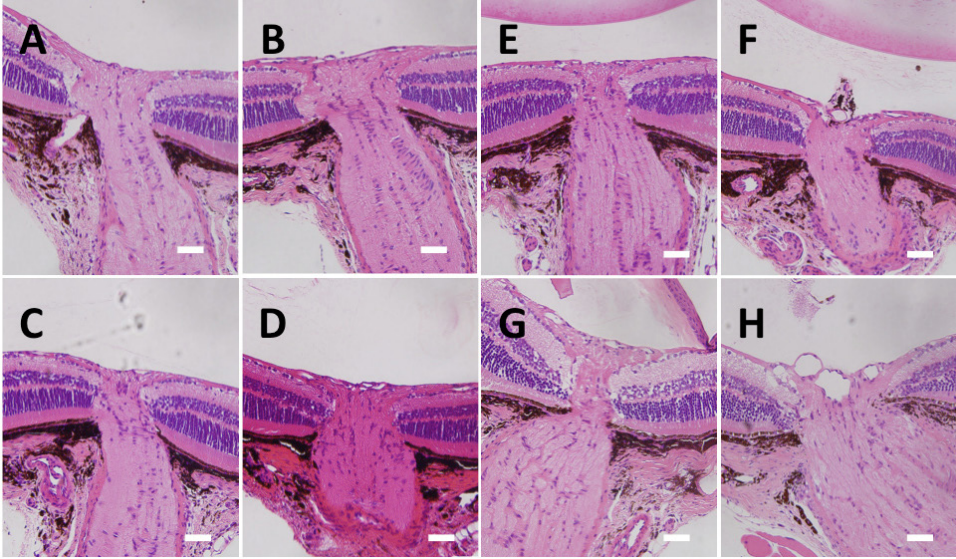
Histological evaluation of the optic disc with various pressure-insult. Pressure-insult dependent optic disc damages were histologically evaluated with the laser-treated eyes. The surrogate values of the pressure-insult are **B**: 53, **D**: 126, **F**: 294 and **H**: 557. Light microscopic images indicate the laser-treated **B**: **D:** **F:** **H:** and the contralateral control (**A**, **C**: **E:** **G:** respectively) eyes. Scale bar indicate 50 μm. These figures indicate that the optic disc cupping become larger as the surrogate value is higher.

Examples of low- and high- magnification images of the experimental retina are shown in Figure 7. Histological analysis revealed that optic nerve degeneration depended on the pressure insult (Figure 8). Four mice were selected, in which two treated eyes of two mice indicated higher surrogate values, with 294 and 557, and the other two treated eyes indicated lower ones, with 53 and 126; thus, these eyes are representative of higher and lower pressure insults. Excavation of the optic disc was observed at the surrogate values 294 and 557 in laser-treated eyes, but not at the surrogate values 53 and 126. There were no obvious changes in the optic disc of contralateral control eyes.

### Regression analysis

**Figure 9:**
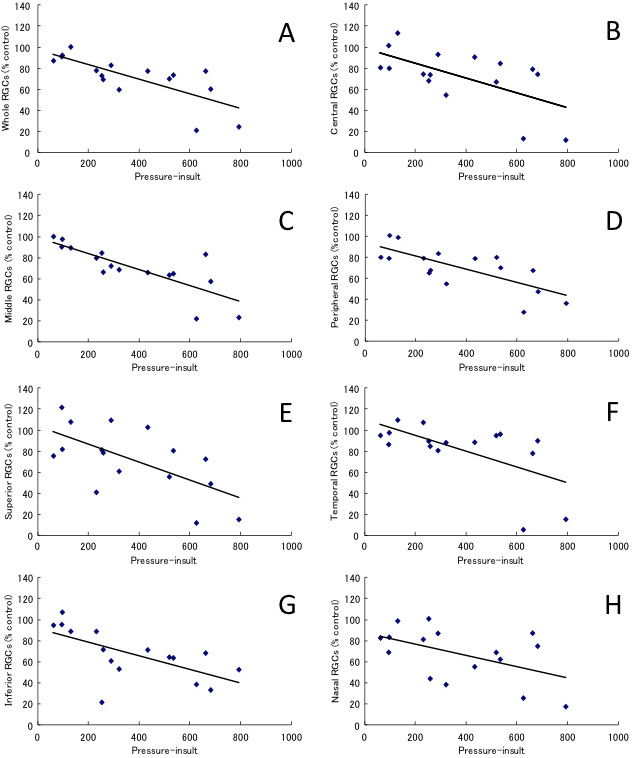
Correlation between the pressure-insult and RGC damage. Regression analysis of **A**: whole retina, **B**: central, **C**: middle, **D**: peripheral, **E**: superior, **F**: temporal, **G**: inferior, and **H**: nasal retinal areas. Linear regression analysis revealed a significant negative correlation between the pressure-insult and the RGC survival rate, calculated as a percentage of the laser-treated RGC density compared with the contralateral control in whole retina (r=0.75, p=0.0008), in the area from optic disc (central; r=0.61, p=0.0119, middle; r=0.08, p=0.0002, peripheral; r=0.73, p=0.0012) and each retinal quadrant (superior; r=0.64, p=0.0082, temporal; r=0.61, p=0.0124, inferior; r=0.63, p=0.0086, nasal; r=0.51, p=0.0427).

The correlation between pressure insult and RGC survival rate was assessed. The pressure insult and the RGC survival rate of the whole retina showed a significant negative correlation (*y*=-0.070*x*+97.2, *r*=0.75, p=0.0008; Figure 9A). Moreover, each area from the optic disc (central [*y*=-0.071*x*+99.1, *r*=0.61, p=0.0119], middle [*y*=-0.077*x*+99.4, *r*=0.80, p=0.0002], and peripheral [*y*=-0.063*x*+93.8, *r*=0.73, p=0.0012]), and each retinal quadrant, (superior [*y*=-0.086*x*+103.8, *r*=0.64, p=0.0082], temporal [*y*=-0.075*x*+110.0, *r*=0.61, p=0.0124], inferior [*y*=-0.065*x*+91.3, *r*=0.63, p=0.0086], and nasal [*y*=-0.055*x*+87.8, *r*=0.51, p=0.0427]) also showed significant negative correlations between the pressure insult and the RGC survival rate (Figure 9B-H). There were no significant differences among the values of the correlation coefficient in seven areas. (p=0.9737) Consequently, our data showed that all retinal areas were evenly damaged. Taken together, the present results demonstrate that induction of chronic IOP elevation in CFP mice results in a significant negative correlation between the pressure insult and RGC survival rate.

## Discussion

One limitation of this study is the ability to control IOP elevation within the range of 20 to 30 mmHg through experimental procedures. Thus, we used a surrogate value of pressure insult indicating pressure damage to each animal. This method will be useful when employing mouse models to screen neuroprotective drugs.

Establishing an ocular hypertension mouse model is a great challenge. To date, laser photocoagulations at the limbus or angle, occlusion of episcleral veins, and microbead injections have been used [19,26-30]. In the present study, laser photocoagulation of the corneal limbus, as described by Aihara et al. [19], was a successful procedure for the induction of chronic IOP elevation in transgenic B6.Cg-TgN(Thy1-CFP)23Jrs/J mice. The time course of mean IOP was similar to that of a previous report [19] (Figure 5). Histological evaluation of the anterior chamber in the laser-treated eye revealed the development of anterior synechia, which probably caused angle closure following aqueous humor outflow reduction and elevated IOP (Figure 4). Additionally, a defined laser trace was observed over the episcleral vein regions similar to a previous report [27], which may augment outflow resistance of aqueous humor. Therefore, our ocular hypertension model is useful as an animal glaucoma model with IOP elevation induced by chronic angle closure. However, there are several concerns in artificially induced ocular hypertension models.

One of the concerns is whether laser photocoagulation causes acute IOP elevation by transient severe inflammation. In this study, the contralateral eyes showed the same number of glaucoma-inducing laser spots around 270° of the limbal circumference as a sham operation, without flattening of the anterior chamber. The sham-operated eyes did not induce IOP elevation (Figure 6). In addition, steroid drops were applied topically for 7 days to prevent inflammation. Thus, it is quite unlikely that laser-induced inflammation cause transient ocular hypertension in our model.

A second concern is IOP fluctuations during the study, especially in the early phase. Our data showed that laser photocoagulation induced chronic elevation of IOP within the first 24 h of the postoperative period and the IOP peaked at 2 days in laser-treated mice. Ocular hypertension was sustained for at least 7 weeks (Figure 4). However, an inherent cure of anterior synechia may have resulted in the recovery of aqueous fluid outflow in 65% of the treated eyes at 8 weeks. Establishment of a model maintaining ocular hypertension over a few months has been problematic. Recently, DBA/2J mice have been used to investigate pressure-dependent RGC damage, but their genetic background is so complicated that RGC damage may be caused by factors other than IOP elevation [9]. Thus, an artificial ocular hypertension model using transgenic or identified mouse strains is desirable. Recently, dexamethasone-induced ocular hypertension mouse models were reported, which indicated sustained ocular hypertension [28]. This mouse model may be more ideal in the future. However, pressure-induced RGC damage can still only be evaluated within 2 months.

A third concern is the transient ischemia caused by acute elevation of IOP. Experimental ocular hypertension models frequently indicate more than 40 mmHg IOP. In our study, 5 of 20 mice showed an IOP of more than 40 mmHg (42–60 mmHg). As the mean systolic blood pressure of C57BL/6J mice, which is the background strain of CFP mice, has been reported to be 93 mmHg [31], the IOP in the present study was well below the systolic pressure. Accordingly, IOP elevation was unlikely to be enough to occlude major retinal vessels. Therefore, we confirmed that cell death occurred due to the magnitude and duration of elevated IOP. In the future, a more ideal ocular hypertension model with a mild IOP elevation of around 20 to 30 mmHg should be developed.

One of the purposes of this study was to investigate the regional differences of RGC loss in the mouse retina. To understand the special susceptibility of RGCs to loss in mouse eyes, it is important to analyze RGC loss in a reproducible and effective way and to investigate the mechanism of RGC loss. Our results showed that the slope of the regression line was located in a narrow range from −0.086 to −0.055 in the central, middle, and peripheral areas of the optic disc and in each of the four retinal quadrant areas. This indicated that all retinal areas were damaged almost evenly by elevated IOP in the CFP mouse eye. Several researchers have reported that peripheral RGC loss is greater than central RGC loss in an inducible elevated IOP rat model [32,33]. Our group previously reported that the superior quadrant of the mouse optic nerve is more susceptible to IOP elevation [20], but the corresponding retinal area for the superior optic nerve has not been clarified in the mouse. A recent report indicated that RGCs in the peripheral area are more susceptible to damage in the microbead-injected ocular hypertension mouse [26]. In this report, RGCs in the peripheral area at 6 weeks after treatment were more damaged than at 2 weeks. Since we evaluated RGC damage at 8 weeks and calculated the correlation of pressure insult and RGC density, regional susceptibility may not have been detected. Laquis et al. could have counted close retinal edges as peripheral, but our method defined the peripheral area as 1800 μm from the optic disc [32]. Besides the different procedures used to induce pressure insult and the methods used to evaluate RGC loss, previous reports have suggested that variation in RGC loss and severity of damage may be caused by regional differences in RGC density within the nerve, genetic variation among individual mice, and possible effects of environmental factors [34].

There are several advantages of our laser-induced ocular hypertension CFP mouse model. The retrograde labeling procedure previously used to visualize RGCs required the injection of exogenous dye into the superior colliculus, which involves a high risk of complications, such as sudden death of the mouse, and induced unknown influences on the results. Additionally, this retrograde method could not label RGCs uniformly compared with RGCs of the CFP mouse [13]. CFP fluorescence of RGCs is intense and stable in paraformaldehyde-fixed tissue. Therefore, the CFP mouse model is easier and represents a more efficient tool for evaluating RGC compared to the previous method.

In contrast, there are also some drawbacks in our model. Although one of the advantages of CFP-expressing cells is that noninvasive assessment may be feasible using a fundus camera, in our procedure with laser treatment, lens opacity sometimes occurred, thereby obstructing fundus photographs. As a consequence, we need to develop a more noninvasive procedure to raise IOP. Previous reports showed a small but significant mismatch between CFP expression in this strain and RGC count with retrograde labeling [13,35]. If there is a difference in susceptibility to stress between CFP-expressing and nonexpressing RGCs, our data may not reflect the real RGC death by ocular hypertension. Moreover, Thy1 expression in RGCs can be downregulated in the setting of stress without cell loss [36]. Thus, our data do not necessarily show that cell death occurred. However, it may be possible to overlook these limitations for the purpose of this model in relation to the screening of neuroprotective drugs.

RGC damage in the present model was also correlated with the magnitude and duration of elevated IOP. Thus, our model has many features resembling chronic human glaucoma. The CFP mouse is available for cross-breeding with various gene-modified mice, and our procedure can induce chronic ocular hypertension in all types of transgenic mice. This has great potential in clarifying the molecular mechanisms of glaucomatous neuropathy.

In conclusion, laser photocoagulation causes a chronic elevation of IOP in CFP mice. The use of CFP mice enabled easy evaluation of pressure-dependent RGC damage without retrograde labeling of RGC. This CFP mouse glaucoma model may contribute to the molecular analysis of neurodegeneration and development of neuroprotective drugs for glaucoma with a great increase in experimental efficiency.
